# An international sepsis survey: a study of doctors' knowledge and perception about sepsis

**DOI:** 10.1186/cc2959

**Published:** 2004-10-14

**Authors:** Martijn Poeze, Graham Ramsay, Herwig Gerlach, Francesca Rubulotta, Mitchel Levy

**Affiliations:** 1 Department of Surgery, University Hospital Maastricht, Maastricht, The Netherlands; 2 Professor and Board of Directors, Atrium Medical Centre, Heerlen, The Netherlands; 3 Professor, Department of Anaesthesia and Intensive Care, Charite Hospital, Berlin Germany; 4 Department of Intensive Care, University Hospital, Leuven, Belgium; 5 Chief of Internal Medicine Intensive Care Unit, Brown University, Providence, RI, USA; 6 On behalf of the European Society of Intensive Care Medicine (ESICM); 7 On behalf of the Society of Critical Care Medicine (SCCM)

**Keywords:** awareness, consensus, definitions, guidelines, intensive care, sepsis

## Abstract

**Background:**

To be able to diagnose and treat sepsis better it is important not only to improve the knowledge about definitions and pathophysiology, but also to gain more insight into specialists' perception of, and attitude towards, the current diagnosis and treatment of sepsis.

**Methods:**

The study was conducted as a prospective, international survey by structured telephone interview. The subjects were intensive care physicians and other specialist physicians caring for intensive care unit (ICU) patients.

**Results:**

The 1058 physicians who were interviewed (including 529 intensivists) agreed that sepsis is a leading cause of death on the ICU and that the incidence of sepsis is increasing, but that the symptoms of sepsis can easily be misattributed to other conditions. Physicians were concerned that this could lead to under-reporting of sepsis. Two-thirds (67%) were concerned that a common definition is lacking and 83% said it is likely that sepsis is frequently missed. Not more than 17% agreed on any one definition.

**Conclusion:**

There is a general awareness about the inadequacy of the current definitions of sepsis. Physicians caring for patients with sepsis recognise the difficulty of defining and diagnosing sepsis and are aware that they miss the diagnosis frequently.

## Introduction

Sepsis is a major cause of death worldwide, with a large impact on mortality in the intensive care unit (ICU). It has been estimated that every day about 1400 patients die in ICUs as a result of sepsis [1].

Recent progress in sepsis research has been able to improve the knowledge about the basic pathophysiological processes of sepsis. However, in daily ICU practice it remains difficult to identify and treat sepsis, and its related conditions, adequately. Concerns remain about the lack of consistent definitions and understanding about sepsis among the global medical community [2,3]. The American College of Chest Physicians and the Society of Critical Care Medicine (ACCP/SCCM) proposed a definition of sepsis and related syndromes in 1991 [4]. Although these definitions were based on expert opinion, the recommendations have not found unequivocal acceptance. However, these definitions have since been used for research purposes investigating new therapeutic modalities, in essentially all intervention trials.

To be able to diagnose and treat sepsis better it is important not only to improve knowledge about definitions and pathophysiology, but also to gain more insight into specialists' perception of, and attitude towards, the current diagnosis and treatment of sepsis. This knowledge is important for the development of strategies to improve consensus in defining sepsis criteria among the intensive care society. Moreover, the introduction of intensivists supporting critical care units has been shown to be associated with improved survival of septic patients [5,6]. Agreement among intensivists, as separate clinical specialists, in terms of their diagnosis of sepsis therefore also needs to be clarified.

Our hypothesis was that although there is good awareness among physicians involved in treating septic patients, a fragmented view of the definitions of sepsis is present. To investigate these hypotheses an international survey was conducted among intensivists and other specialists involved in the diagnosis and treatment of sepsis.

## Materials and methods

In an international survey 1058 physicians were interviewed for this study; they were interviewed after a random selection of 1100 physicians in Europe and the USA. Of these, 756 physicians were interviewed in France (*n *= 150), Germany (*n *= 155), Italy (*n *= 150), Spain (*n *= 151) and the UK (*n *= 150). A further 302 physicians were interviewed in the USA. In each country equal numbers of intensive care and other specialists were interviewed. The specialist physicians included anaesthesiologists, cardiologists, endocrinologists, internists, nephrologists, pulmonologists, surgeons and emergency room physicians. The intensivists had to spend 50% or more of their time treating adults in the ICU, had to treat on average five or more ICU patients per month, had to treat two or more adult sepsis patients per month on average, and had to have worked for 2 years or more in the ICU. Otherwise they were classified as other physicians. The other specialists were also involved in the treatment of patients with sepsis, although on a less regular basis (fewer patients). They had to spend 10% or less of their time treating adult patients in the ICU and had to have been in practice for at least 2 years. It was intended that physicians spending between 10% and 50% of their time in the ICU should be excluded, but no physicians fulfilled this exclusion criterion. The study was conducted from November to December 2000. A recent study has shown a reduced mortality in patients with septic shock [7]. However, it was performed before the results of the present study were available. The survey was performed by telephone interview using trained staff of Yankelovich Partners. We list the questions asked in additional file 1. All questions were grouped into three categories based on a model describing behaviour framework [8]. To implement sepsis definition guidelines effectively, first the physician's awareness of the problem should be raised, then agreement on the problem should be reached and finally the ability to implement the definition guidelines should be present.

### Statistics

The data for this study are presented as means ± SEM or as percentages. Data were analysed with Student's *t*-test or χ^2 ^testing. *P *< 0.05 was considered statistically significant.

The margin of error for the total group of physicians in this study was 3.0%, on the basis of the combined error values of all questions combined.

## Results

### Respondent profile

Most physicians (83%) were male with an average age of 44.2 ± 0.3 years. The majority (57%) of these physicians were working in a non-teaching hospital. There was no difference between the intensivists interviewed and the other physicians with respect to gender, age distribution, percentage working in teaching hospitals, and percentage of practice based in hospital (Table 1). The intensivists worked on an average 77.2 ± 0.95% of their time in the ICU. The number of adult patients treated in the ICU per month by the intensivists was 60 ± 3; of these 16.5 ± 0.9 were septic patients. The intensivists had worked for 11.6 ± 0.3 years after residency on the ICU. Of the other physicians, interviewed 120 (23%) were anaesthesiologists, 26 (5%) cardiologists, 26 (5%) endocrinologists, 83 (16%) internists, 18 (3%) nephrologists, 48 (9%) pulmonologists, 32 (6%) surgeons, 119 (23%) emergency room physicians, and 57 (11%) oncologists. These physicians worked 4.0 ± 0.3% of the time on the ICU and had been 13.5 ± 0.4 years in practice since residency.

### Awareness of the problem of sepsis

Three-quarters (767 of 1058) of all interviewed physicians agreed (strongly or somewhat) that sepsis is a leading cause of mortality compared with other conditions in intensive care. Of the intensivists, 78% considered sepsis as the leading cause in comparison with 67% of other physicians (*P *< 0.0001). Nine in ten (934 of 1058) physicians agreed (strongly or somewhat) that sepsis is a significant financial burden on the health care system in their country. Among all physicians, 88% (937 of 1058) considered sepsis among the most challenging conditions that a doctor can treat. Two in five physicians (420 of 1058) had the impression that the incidence rate of sepsis has increased 'steadily' to 'dramatically' over the past 5 years, whereas 48% said that it remains stable. Two-thirds (285 of 420) thought that this increase is either 'extremely serious' or 'very serious'. Of the physicians surveyed, 77% reported the following major factors involved in this increase: an increased resistance of bacteria to antibiotics, an increased number of immuno-compromised patients, and a higher survival chance of post-surgical patients and patients with serious pathology.

A majority (656 of 1058, 62%) of physicians believed that their definition of sepsis is commonly accepted within their speciality. More than four in five (905 of 1058, 86%) physicians agreed (strongly or somewhat) that the symptoms of sepsis can easily be misattributed to other conditions. There was concern (ranging from 'somewhat' to 'extremely concerned') about the lack of a common definition for sepsis in 67% (708 of 1058) of the physicians. Of the physicians who were concerned about the lack of a common definition, 83% (199 of 708) stated that it is at least somewhat likely that the diagnosis of sepsis is missed. This figure was 53% (29 of 350) for the physicians who were not concerned about the lack of a common definition for sepsis. Although physicians are divided over whether the lack of a common definition for sepsis hinders proper diagnosis, they are not divided over whether a common definition would be a significant step towards better treatment.

### Agreement on definitions of sepsis

In general, physicians' definitions of sepsis were fragmented. When defining sepsis, only 22% (114 of 529) of the intensivists and 5% (26 of 529) of the other physicians gave the definition of the ACCP/SCCM consensus statement (*P *< 0.0001). Fewer than one-fifth (17%) of the physicians agreed on any one definition for sepsis, and six different definitions were mentioned by at least 1 in 10 physicians. This was not different between intensivists and other physicians. Moreover, physicians were divided as to whether sepsis is a systemic response (46%, 490 of 1058) as opposed to a syndrome (36%, 380 of 1058). One in ten physicians (103 of 1058), of both the intensivists and the other physicians, said that sepsis is a disease.

Among physicians, 71% (751 of 1058) said that fever is a sign or symptom that must be present to diagnose sepsis rather than any other factor. Aside from fever, no one symptom was listed by a majority of physicians as a sign or symptom that must be present to diagnose sepsis. Tachycardia was only cited by 29%, leukocytosis or leukopenia by 20%, hypothermia by 14%, and tachypnoea by 9% of physicians.

### Ability to diagnose sepsis and communicate about sepsis

Four in five physicians (911 of 1058) agreed (strongly or somewhat) that patients need better monitoring to diagnose sepsis at the earliest possible stage. In addition, 84% (890 of 1058) agreed (strongly or somewhat) that patients are often treated too late to reverse the onset of sepsis. According to the physicians, 46% of sepsis deaths are recorded as death by other diseases rather than death by sepsis. Bacterial culture results ranked as the most effective method for diagnosing sepsis by physicians; 80% found bacterial cultures either 'extremely' or 'very effective'. The second most effective method for diagnosing sepsis was haemodynamic monitoring. A significantly greater percentage of intensivists (74%, 393 of 1058) than the other physicians (66%, 350 of 1058) ranked haemodynamic monitoring as either extremely or very effective (*P *= 0.002) for diagnosing sepsis. Two-thirds (65%, 684 of 1058) of physicians agreed that a physical examination of symptoms is an effective method.

When speaking to the patients' relatives, 81% (858 of 1058) of physicians agreed that communicating a diagnosis of sepsis to the families of patients with sepsis is difficult. Therefore, more than four in five (85%, 899 of 1058) physicians said that they describe sepsis to patients' relatives as a complication arising from an underlying condition, as opposed to 10% who said they describe the diagnosis as sepsis.

## Discussion

In the present age of intensive care, sepsis remains responsible for a considerable number of deaths in critically ill patients. This disease has a major impact on both health care and society resources. Despite an increased understanding of sepsis, so far no information has been presented about physicians' perception and knowledge of sepsis. This international survey was therefore conducted among physicians involved in treating septic patients.

One of the main findings of this study is that there is a general awareness of the importance and impact of sepsis among the physicians interviewed. A vast majority of physicians consider sepsis a leading cause of mortality. Moreover, the physicians agree that sepsis is a commonly encountered condition with an increasing incidence. Two recent reviews summarised the published studies on the incidence and mortality rates reported for sepsis. In a review by Brun-Buisson [9], 25% of patients on the ICU develop sepsis, with incidence rates varying from 45 in 1000 hospital admissions to 494 in 1000 ICU admissions. In a review by Matot, sepsis occurred with a mean frequency of 22.4% [1]. In both reviews a clear division between definitions of sepsis and severe sepsis or septic shock was used. In the review by Brun-Buisson an additional 10–15% of patients developed septic shock [9]. In practice, however, a majority of physicians agree that it is at least somewhat likely that the diagnosis of sepsis is being missed frequently.

One of the remarkable findings of this study is the lack of agreement on the definition of sepsis. A new set of definitions was proposed by the consensus conference of the ACCP/SCCM in 1992 [4] to improve the bedside recognition of sepsis, to permit early intervention and to differentiate infectious from non-infectious conditions. However, only a small percentage of physicians report the ACCP/SCCM criteria for the definition of sepsis. Not more than one-fifth agree on any one definition. This is consistent with the fact that a majority of physicians were concerned that there is no common definition of sepsis and a large proportion of physicians (for non-intensive care physicians even 41%) believe that other physicians within their speciality define sepsis differently from themselves. This perceived lack of a common definition might also explain why a significant number of physicians believe that sepsis is missed as a diagnosis. Indeed, the recommendations from the International Sepsis Forum recognise that in the past different definitions of sepsis were used interchangeably, which led to confusion [10].

When looking at the precise criteria that must be present according to the physicians interviewed, a wide variety of signs and symptoms were given. The one factor most frequently quoted was fever; the second most frequent answer was hypotension. This is of interest, given the fact that intensivists, in this survey, considered themselves extremely knowledgeable about the definition of sepsis and in the distinction between sepsis, severe sepsis and septic shock. Both the use of only one criterion and the use of hypotension are not at all consistent with the consensus definitions established in 1992 [4]. This misunderstanding with regard to the consensus criteria is consistent with the perception, among most physicians surveyed, of a lack of clear definitions for sepsis.

The lack of agreement on the definitions of sepsis criteria has an influence on the ability of physicians to diagnose and communicate about sepsis. The physicians in this survey were not content about the diagnostic tools they have for the diagnosis of sepsis. Most physicians agreed that better monitoring tools are needed to diagnose sepsis at the earliest possible time. Although a large percentage of physicians surveyed considered bacterial cultures and haemodynamic monitoring very effective for diagnosing sepsis, they also reported a high degree of interest in the investigation of other, more sensitive tools.

Another aspect of this survey was the differences found between intensivists and other specialists with less involvement in ICU care, indicating a difference in patient numbers with sepsis. Recent studies investigated the effects of specialised ICU staffing on outcome [[5,6,11], 12]. The results of these studies suggested that the presence of intensive care physician staffing is associated with a decreased length of ICU stay and with decreased costs, complications and mortality. However, it remained relatively unclear whether the institution of specialised ICU staffing had its effects on agreement, awareness and ability to diagnose sepsis. This survey showed that in general the intensivist seems to be more aware of issues involved for critically ill patients with sepsis. More intensivists consider sepsis a leading cause of mortality and a significant financial burden on the health care system. Moreover, they more frequently have the impression that the incidence is increasing. However, although awareness seems to be higher in specialised ICU staff, agreement on the definitions of sepsis is just as scattered as with non-ICU specialists. As a consequence the ability of intensivists and other specialists to diagnose sepsis is more or less comparable. Moreover, the ability of physicians to communicate the diagnosis of sepsis to the patients' relatives is equally problematic. Two conclusions can be drawn from this survey, despite the limitations of a telephone survey. First, many doctors cannot define sepsis in accordance with the previously published consensus criteria. Second, sepsis is perceived as a leading cause of death in ICUs. The incidence of sepsis is high, and in addition physicians believe that the diagnosis of sepsis is often missed. This survey lends support to the idea that definitions of sepsis should be reviewed and that education is required, for both physicians and the public, for a better standardisation of clinicians' definition and diagnosis of sepsis.

## Key messages

• 	The current awareness of physicians concerning the impact which sepsis has on resources is widespread.

• 	Physicians are concerned that lack of agreement on the definitions of sepsis may lead to underestimating of the incidence of sepsis.

• 	The lack of agreement on the definitions of sepsis criteria has its influence on the ability of the physicians to diagnose and communicate about sepsis.

## Competing interests

The author(s) declare that they have no competing interests.

## Abbreviations

ACCP = American College of Chest Physicians; ICU = intensive care unit; SCCM = Society of Critical Care Medicine.

## Supplementary Material

Additional File 1 A PDF file containing a list of questions from the international sepsis survey. Click here for file

## Figures and Tables

**Table 1:** Respondent demographics

Respondent profile	Intensivists	Other specialists	*P*
Number	529	529	
Gender (% female)	14	20	0.2
Age, years (mean ± SEM)	43.8 ± 0.4	44.6 ± 0.4	0.7
Working in teaching hospital (%)	43	42	0.5
Percentage of practice based in hospital			
Less than 30%	1	6	
30–50%	2	3	
50–70%	8	5	
More than 70%	88	85	
Unknown	1	1	0.4

Comparison of respondent demographics was by χ^2 ^or Student's *t*-test.
