# Supplementary material for: An international sepsis survey: a study of doctors' knowledge and perception about sepsis
Source: Crit Care. 2004 Oct 14;8(6):R409–13. doi: 10.1186/cc2959 (PMC1065059; doi:10.1186/cc2959)
Supplement: Additional File 1 — A PDF file containing a list of questions from the international sepsis survey. [file cc2959-S1.pdf]

Appendix 1. Abstracted question form international sepsis survey

Sepsis – The challenge, the dilemma

- *How knowledgeable are you about sepsis, its treatment, and the complications associated with it?* Are you: Extremely knowledgeable – Very knowledgeable – Somewhat knowledgeable – Not too knowledgeable – Not at all knowledgeable

- *Agree or disagree ?*: Strongly agree – Somewhat agree – Somewhat disagree – Strongly disagree – Don't know
  Sepsis is a leading cause of mortality compared to other conditions
  Sepsis treatment is one of the unmet needs in critical care today
  Sepsis is a significant burden on the healthcare system in my country
  Sepsis is among the most challenging conditions a doctor can treat

- *Do you believe that your definition of sepsis is commonly accepted within your speciality or do you believe that other physicians within your speciality may define sepsis somewhat differently than you do ?*: Commonly accepted – Define somewhat differently – Not sure

- *Agree or disagree ? Medical specialists such as yourself in your country are aware of the differences between sepsis/severe sepsis and septic shock ?* Strongly agree – Somewhat agree – Somewhat disagree – Strongly disagree – Don't know

- *Agree or disagree ?*: Strongly agree - Somewhat agree - Somewhat disagree - Strongly disagree - Don't know
  There is an accepted definition of sepsis in my country
  The pathogenesis of sepsis is not completely understood
  The symptoms of sepsis can be easily misattributed to other conditions

- *How concerned are you that there is no common definition of sepsis within the medical profession ?* Are you: Extremely concerned - Very concerned - Somewhat concerned - Not very concerned - Not at all concerned - Not sure.

- *Because there is no common definition of sepsis, how likely is that the diagnosis of sepsis is being missed ?* Is it: Extremely likely - Very likely - Somewhat likely - Not very likely - Not at all likely - Not sure

- *Agree or disagree ? A common definition for sepsis for the global medical community would be a significant step toward better treatment toward better treatment ?* Strongly agree - Somewhat agree - Somewhat disagree - Strongly disagree - Don't know

<u>Definitions - The many shades of sepsis</u>

- *Based upon everything you know, how do you define sepsis ?* Multiple answers allowed, Unaided, coded responses.

- *Do you view sepsis as a .... ?* Systemic response - Syndrome - Disease - Condition - Disorder - Other - Not sure

- *What causes sepsis to occur ?* Multiple answers allowed, Unaided, coded responses.

- *What are the signs and symptoms that must be present to diagnose sepsis ?* Multiple answers allowed, Unaided, coded responses.

<u>Prevalence and impact of sepsis</u>

- *Thinking of the past year, what percentage of your patients were either diagnosed or suspected to have sepsis ?*

- *What would you say is the average age of your sepsis patients ?*

- *What percentage of patients who develop sepsis die as a result ?*

- *Thinking about the last 5 years, would you say that the incidence rate of sepsis has been ... ?* Increasing dramatically - Increasing steadily - Remaining the same - Decreasing steadily - Decreasing dramatically - Not sure.

- *Why do you say the incidence rate is increasing? Is it due to a(n)*
  Increased resistance of bacteria to antibiotics
  Increase in immunocompromised patients

Higher survival chances of post-surgical patients and patients with serious pathologies

Change in population composition

Increase in organ transplant populations

Increased number of surgeries

Other

- *How serious of a problem do you think the rise in the incidence of sepsis is ?* Do you think it is ..: Extremely serious - Very serious - Somewhat serious - Not very serious - Not at all serious.

- *What percentage of patient deaths from sepsis do you believe are recorded as death by the original condition rather than death by sepsis ?*

Diagnosing and communicating sepsis

- How effective are the following methods for diagnosing sepsis ? (scale 1-5, 1 not at all effective and 5 extremely effective)

  Bacterial culture

  Haemodynamic monitoring

  Physical examination of symptoms

  Blood count

  Antibiogram

  Arterial blood gas

  Other laboratory examinations

  Hepatic function

Biological marker

Protein C levels

TNF test

IL-6 test

IL-1 test

- *Agree or disagree?* Strongly agree - Somewhat agree - Somewhat disagree - Strongly disagree - Don't know

  Patients need better monitoring in order to catch sepsis at the earliest possible stage

  Patients are often being treated too late to reverse the onset of sepsis

  Families of sepsis patients find it difficult to understand sepsis

- *Which do you do more often - describe the patient's diagnosis as sepsis or do you describe the patient as having sepsis as a complication from the original condition ?* Diagnosis as sepsis - Diagnosis as complication - Not sure

Treating sepsis

- *Overall how satisfied are you with the treatment options currently available for treating sepsis ?* Are you ..

  Extremely satisfied - Very satisfied - Somewhat satisfied - Not very satisfied - Not at all satisfied - Not sure

- *Agree or disagree ? The current treatment options for sepsis are not adequate ?* Strongly agree – Somewhat agree – Somewhat disagree – Strongly disagree – Don't know

- *Agree or disagree ? Doctors are eager for a breakthrough in treating sepsis ?* Strongly agree – Somewhat agree – Somewhat disagree – Strongly disagree – Don't know

- *Which of the following therapies do you yourself use to treat these sepsis patients ?*

Antishock/organ support therapy:
Stabilize/reverse cardiovascular and metabolic disturbances
Proper fluid/volume resuscitation
Vasopressors
Inotropes
Monitoring in the ICU
Ventilation

Invasive surgical/radiological therapy:
Removal of infected prostheses of catheters
Abscess drainage
Debridement of necrotic tissue

Antibiotics
Steroids
Depend upon the patient
Other
